# Variance in Centrality within Rock Hyrax Social Networks Predicts Adult Longevity

**DOI:** 10.1371/journal.pone.0022375

**Published:** 2011-07-27

**Authors:** Adi Barocas, Amiyaal Ilany, Lee Koren, Michael Kam, Eli Geffen

**Affiliations:** 1 Department of Zoology, Tel Aviv University, Tel Aviv, Israel; 2 Department of Comparative Biology and Experimental Medicine, Faculty of Veterinary Medicine, University of Calgary, Calgary, Canada; 3 Desert Animal Adaptations and Husbandry, Wyler Department of Dryland Agriculture, The Jacob Blaustein Institutes for Desert Research, Ben Gurion University of the Negev, Beer Sheva, Israel; University of Manitoba, Canada

## Abstract

**Background:**

In communal mammals the levels of social interaction among group members vary considerably. In recent years, biologists have realized that within-group interactions may affect survival of the group members. Several recent studies have demonstrated that the social integration of adult females is positively associated with infant survival, and female longevity is affected by the strength and stability of the individual social bonds. Our aim was to determine the social factors that influence adult longevity in social mammals.

**Methodology/Principal Findings:**

As a model system, we studied the social rock hyrax (*Procavia capensis*), a plural breeder with low reproductive skew, whose groups are mainly composed of females. We applied network theory using 11 years of behavioral data to quantify the centrality of individuals within groups, and found adult longevity to be inversely correlated to the variance in centrality. In other words, animals in groups with more equal associations lived longer. Individual centrality was not correlated with longevity, implying that social tension may affect all group members and not only the weakest or less connected ones.

**Conclusions/Significance:**

Our novel findings support previous studies emphasizing the adaptive value of social associations and the consequences of inequality among adults within social groups. However, contrary to previous studies, we suggest that it is not the number or strength of associations that an adult individual has (i.e. centrality) that is important, but the overall configuration of social relationships within the group (i.e. centrality SD) that is a key factor in influencing longevity.

## Introduction

Group living occurs in many animal taxa, including invertebrates, fishes, birds and mammals [1], and is thought to offer protection against predators and to increase foraging efficiency, two factors which may have favored the evolutionary transition from solitary to group foraging [2]. Empirical studies of social mammals have shown that living in groups also has significant consequences for reproductive success. Larger groups offer better protection against infanticide (e.g. [3]) and kleptoparasitism (e.g. [4]). Group size has been shown to correlate with reproductive success in a number of plural breeders (e.g. [5]) and a non-linear relationship was demonstrated in other systems, with the largest and smallest groups showing the lowest reproductive success [6]. It has been suggested by Clutton-Brock et al. [7] that the variation between group size and reproductive success is dependent on social system. In species that rely on helpers, large groups increase reproductive success, but reproductive success is reduced in large groups in species that lack helpers. Furthermore, the actions of an individual, especially a dominant one, may have significant consequence on all others in the group [8]. For example, eviction of a specific individual may increase resources and survival of all other group members, but also provide additional benefits to the dominant animals (e.g. reduce competition and increase fitness). Not only fitness is associated with group size, mortality is as well. For example, in lions (*Panthera leo*), the number of females in a group has a negative effect on adult mortality [6]. Studies in rodents, however, have revealed an opposite effect, with females from larger groups showing reduced fitness [9], [10]. Males in polygynous systems are subjected to intense competition with other males, which may result in lower male survival due to the risk of injury and susceptibility to starvation (reviewed in [8]). Still, limited evidence is available on how group size and social characteristics might shape the survival probabilities and longevity of the group members.

In recent years, biologists have realized that the size of a group is not the only factor that increases group members' survival, but that within-group interactions also have significant implications. Several recent studies have demonstrated that the social integration of adult females is positively associated with infant survival, an important component of the variation in female lifetime fitness [11]–[13]. Another key component, female longevity, is similarly affected by the strength and stability of social bonds [14].

We analyzed social bonds in a wild rock hyrax (*Procavia capensis*) population using 11 years of accumulated behavioral data collected in Ein Gedi Nature Reserve, Israel. The group-living rock hyrax is a plural breeder, with the animals living in social units composed mainly of females. Social hierarchy is not steep among females, with all females reproducing yearly [15], [16]. Some forms of communal care for young such as babysitting, pup protection from both resident and alien males, and guarding against predators, are common [15]–[18]. Male hyraxes disperse as juveniles, between the ages of 16 and 30 months, and often remain in the home range near the natal group area [19]. It is currently unknown whether dispersing hyraxes are more vulnerable to predation and have lower chances of survival due to their solitary life and inter-male aggression.

Social network theory provides a framework for quantifying individual and group-based parameters of a specific social structure [20]. The approach we present here uses weighted associations between animals (ties or edges), calculated as the proportion of days they were observed together out of the total number of days each animal was seen. This ‘association index’ serves as a basis for calculation of various measures describing the centrality of each individual within its social group. Our study examined social factors that influence adult survival in groups of the rock hyrax. We tested for association between individual and group social parameters and longevity, taking into consideration group size. In addition, we used mark-recapture analysis based on the Akaike Information Criterion (AIC [21]) model selection approach in order to determine whether solitary adult males show reduced survival probabilities compared to individuals living in groups.

## Materials and Methods

### Ethics Statement

This study was conducted under annual permits from the Israeli Nature and Parks Authority for capturing, handling and sampling the hyraxes at the Ein Gedi Nature Reserve (2000/8871, 2001/8871, 2002/14674, 2003/14674, 2004/17687, 2005/17687, 2007/27210, 2008/31138, 2009/32871).

### Field protocols

We have been conducting a long-term study at the Ein Gedi Nature Reserve (31°28′N, 35°24′E) since 1999. Our study sites are located in two deep gorges, David and Arugot, which constitute part of the reserve located west of the Dead Sea, Israel. During each field season, beginning in March and varying in length from three to six months, rock hyraxes were trapped and observed daily. We used Tomahawk live box traps, which were placed in secure shady spots, and baited with cabbage and kohlrabi. Since rock hyraxes are diurnal, traps were opened for a fixed period of time during the morning. The trapped animals were anaesthetized with ketamine hydrochloride (0.1 mg/kg), fitted subcutaneously with microchips (DataMars SA), as well as with either an ear tag (for pups and juveniles) or a light collar (weighing<5 g), with tags attached to identify them for observation from a distance. Captured hyraxes were sexed, weighed and measured. Animals were allowed 90 to 150 minutes of recovery after anesthesia. Recaptures were not anaesthetized and released immediately after weighing and hair sampling.

Animals captured as pups were of known age. Rock hyraxes breed synchronously [22], so pups were aged from March 1 of the year of capture, and one year old juveniles were aged from March 1 of the previous year. We used linear regression to predict age of all other individuals that were not captured as pups, as we have previously found that body weight (log-log transformation) was the best predictor of hyrax age [18]. We used the following equations to estimate age of males (Log (Age) = 2.3250903+1.3498142*Log (BW), r^2^_69_ = 0.934, P<0.0001) and females (Log (Age) = 2.3711934+1.4997657*Log (BW), r^2^_107_ = 0.930, P<0.0001). These equations were calculated based on a large sample of animals for which the true age was known (i.e., were trapped as pups). Age of death was calculated in years to the last season in which an individual was seen or captured alive.

Hyrax activity was observed daily during the field season using 10×42 binoculars and a telescope with 50–100× magnification (C5 spotting scope, Celestron, USA). Observations were conducted in the morning from first light to noon, when hyraxes in Ein Gedi retreat to their shelters. Each day, a focal group was randomly chosen and followed [23]. One observer scanned using binoculars, locating individuals, while the other used a telescope to identify the animals using their marks. Using this method we were able to record multiple social interactions within a group, including interactions of non-group members in the same area. Hyraxes spend most of their time foraging and resting (e.g. [24]), making it easy to follow multiple individuals simultaneously. However, we could not measure the exact duration of all pairwise social interactions due to the limitations of following up to 10 individuals simultaneously and due to limited visibility caused by rocks, trees, and bushes. Therefore, we used a resolution of one day to define if two individuals were seen in social interaction, regardless of the duration of interaction. Every year 95%±0.5 of group members were marked, facilitating minimal bias in recording the social structure of each group. The few social interactions that included any unmarked individuals were excluded from the analysis. Overall, we compiled data from 255 observation days (about 1,500 hrs) at the Arugot site (2000–2004, 2007–2009; 39±6.7 adults per year) and 117 days (about 700 hrs) at the David site (2002–2004, 2007–2009; 29±3.4 adults per year), and recorded 932 and 485 events of positive interactions, respectively [18], . We regard positive interactions as any of those that included physical contact (i.e. huddle or hole up together in a sleeping burrow), or those that show coordinated activity (move together in close proximity, and sit beside one another). We counted the number of days in which social interactions between each pair of individuals occurred over the determined period of observations (e.g. one year), and used this count data to construct social networks. The one-day resolution was used in order to equalize observation time among individuals [27]. We excluded from this analysis observations of foraging on trees, which may force animals into close proximity only due to the physical constraints of tree climbing and not due to social context [28]. We also excluded all agonistic interactions from our network analysis, as these did not reflect a positive association between individuals. Agonistic behavior was reflected in a combination of typical actions and postures, such as displaying the large incisors, growling, grinding molars, snapping, and chasing and biting others [25], [28].

### Network analyses

Our network analysis was set by three steps: 1) Construction of networks based on the observation data and statistically testing for presence of long-term associations between individuals. Only networks that are significantly different from random networks (i.e. describe long-term relationships between individuals) were used for further analysis. 2) Assignment of individuals into groups within each network, using an objective algorithm. 3) Calculation of individual and group social parameters within each group.

**Figure 1:**
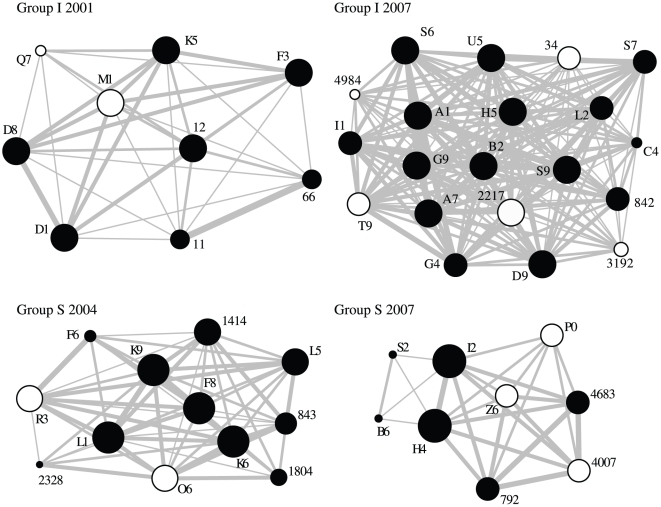
Examples showing networks with low (group I 2001, group I 2007) and high centrality SD (group S 2004, group S 2007). Full and empty circles represent females and males, respectively. Tie width (i.e. connection between nodes indicated by circles) is proportional to the values of the association index. Node size is proportional to centrality.

We used the program SocProg 2.4 [29] to generate an association matrix for each site in each season. We used a simple association index, which is a ratio of the number of days in which two individuals were seen together out of the total number of days they were each observed. We then randomized within samples, to determine whether certain individuals had associations that differed from random values. This randomization procedure tests for long-term companionships or avoidances [30]. We ran a progressively increasing number of permutations (maximum 10,000) until the P-value was stabilized. This analysis was applied to 13 social networks with sufficient observation sessions (>10 sessions per area). We used observation data collected during 2000–2004 and 2007–2009 at the Arugot site, and during 2002, 2004 and 2007–2009 at the David site (Fig. 1). A total of 11 out of the 13 networks, eight at Arugot and three at David, showed significantly different association values from the randomly generated networks (P<0.05), and only these networks were kept for further analysis.

In each site, there were usually two groups of hyraxes each year and some solitary bachelor males. In order to analyze the interactions within each group we first had to define which individuals belong to each group. While for some individuals that is straightforward, others were seen interacting with group members only part of the time and therefore an objective algorithm was required to assign individual hyraxes to groups. We chose the weighted clique percolation method (CPMw), a community detection algorithm, to define groups within our populations [31], [32]. This algorithm builds up network communities by joining together individuals that share strong associations. The software CFinder (version 2.0.1) was used for running the CPMw algorithm [32]. We used three age categories [16], [18]: pups (≤1 year old), juveniles (older than 1 but younger than 2 years old), and adults (≥2 years old). All marked adults and juveniles (≥1 years old) in each season were included in the social network analysis. Pups were excluded. The groups defined by the algorithm within each population were used for further analysis, including measures of centrality and group size. While group membership changes over the years as individuals die, leave or join, some level of stability remains, meaning that groups in consecutive years are not fully mixed. This may resemble a human sports team where some players join or leave each year but the core of the group remains.

We used the social network analysis program Ucinet (version 6.258; [33]) to calculate, for each group and year, the following network variables: 1) Individual strength centrality (or weighted degree centrality, hereafter centrality) is the sum of all association indices that each individual had in the network. We used the extension of Freeman's degree centrality [20] to weighted networks as the measure of an individual's centrality within the network [34]. 2) Group strength centrality SD is the standard deviation in strength centrality within the group. 3) Individual power [35] is an extension of the Freeman's centrality measure, which takes into consideration each node's neighbors and how connected they are, and calculates centrality in an iterative manner. 4) Group power SD is the standard deviation in power within the group. 5) Individual information centrality is a measure based on the harmonic mean length of paths reaching each individual [36]. Group information centrality SD is the standard deviation in information within the group. 6) Distance based cohesion (or compactness; [37]) is the harmonic mean of all path lengths within a group. 7) Network centralization (or global centrality; [38]) is a measure of the degree to which an entire network is focused around a few central nodes. The last two network measures are not individual measures but associated with the whole group network. The chosen parameters allowed us to test the effect of different individual and group social measures on longevity.

Social network variables for animals present in multiple years were averaged over all relevant years, starting from the age of two. For example, if a hyrax died at age four after belonging to the same group over its lifespan, its mean group strength centrality SD was the mean of its group strength centrality SD when it was two, three and four years old.

### Agonistic behavior

Although our analysis focused primarily on positive interactions, we have also explored the effect of negative (i.e. agonistic) interactions because some aggressive conflicts end up in severe injuries or death of adults. Agonistic interactions were defined above and following our previous publications on social rank [16], [18], [25]. We calculated two individual measures reflecting aggression within a group: individual and group aggression rates. Individual aggression rate is the number of days we observed a focal individual involved in agonistic interactions controlled for group size and the number of days it was observed as a group member. Group aggression rate is the number of days we observed agonistic interactions within a group controlled for group size and the number of days the group was observed during the period the focal animal was a group member. We controlled for observation time and group size because these variables correlated with number of agonistic interactions within group and by individual (r^2^ = 0.937, F_2,26_ = 193.3, P<0.0001 and r^2^ = 0.489, F_2,26_ = 12.4, P = 0.0002, respectively).

### Mark-Recapture Analysis

We constructed an encounter history for both sites in which hyraxes were trapped and observed (Arugot site: 1999–2009; David site: 2002–2009). Hyraxes captured as pups were put in a separate age class to animals caught as juveniles or adults. Animals belonging to distinct social groups were assigned to their respective group following the CPMw results (see social network analysis section). Solitary males were assigned to a separate group in each population.

We used the program MARK [39] for the mark-recapture analysis. To use data from different sources, and increase the accuracy of hyrax survival estimates, we chose the Barker model [40], which allows the use of live recaptures, dead recoveries and live resights. This model also allows the inclusion of observations made between recapture sessions. It enabled us to include marked animals, which on some occasions were not recaptured but were seen in the research area. Animals that were reported dead (body found) or their collars were recovered were included in the analysis as dead recoveries. During our study, we had only two cases in which collars have fallen off, out of 177 collared individuals (1.1%), allowing us to assume a found collar to belong to a dead animal. Furthermore, since we trap each year more than 80% of the study population and all individuals are double-marked with microchips, we could verify if animals without collars are new or ones that have lost their collars. The cause of mortality for most animals was not known. Our preliminary analysis did not show any evidence that recapture probability is affected by trapping effort [26].

Traditional survival and recapture estimation models, the basic Cormack-Jolly-Seber (CJS) models, mainly use two different parameters: phi (apparent probability of survival) and p (probability of recapture). We used the Barker model, which is a more suitable model for our data (i.e. recapture and resight) and for modeling survival and emigration. The Barker model extends the CJS by using the following additional parameters [40], [41]:

*S*_i_ - the probability that an animal alive at time i is alive at time i+1
*p*_i_ - the probability that an animal is captured at time i, given that it is alive and at risk of capture at time i
*r*_i_ - the probability that an animal that dies in the interval between i and i+1 is found and has its band (or collar) reported
*R*_i_ - the probability that an animal alive in i+1 is resighted alive during the interval between i and i+1
*R′*_i_ - the probability that an animal that dies in the interval between i and i+1 is resighted alive in this interval before it dies
*F*_i_ - the probability that an animal at risk of capture at i is at risk of capture at i+1
*F′*_i_ - the probability that an animal not at risk of capture at i is at risk of capture at i+1

Goodness-of-fit of a global model was assessed using the median ĉ procedure, which is based on a logistic regression of simulated deviance values for progressively higher c values, where c is the over-dispersion measure [42]. Each ĉ value was calculated using 20 replicates. The over-dispersion statistics were found to be low (Arugot site: ĉ ± SE = 1.357±0.02, David site: ĉ = 1.097±0.01). We used the ĉ values to correct our model selection for the effects of small sample size and over-dispersion (QAICc; [21]). To compare different models from a candidate set we used the Akaike Information Criteria (AIC). The relative likelihood of each model was estimated using normalized AIC weights (W_i_). Each parameter was modeled separately following the model selection procedure [43]. Fidelity and return rates were modeled as time-dependent, constant, or dependent on group type (male or mixed groups, Table S1). Resights (R) and resightings before dead recovery (R′) probabilities were allowed to vary over time, be constant, or vary over observation years. Dead recovery (r) was modeled as time-dependent or constant. We examined the fit of models for recapture rate (p) using time-dependence, age-dependence (i.e. two age classes) and group-dependence. After obtaining the optimal model (lowest QAIC), we proceeded to model survival (S). Age-dependence, differential survival between groups, and time-dependence in pup and male survival were examined. Model nomenclature followed the format in [44]. The derived survival estimates were obtained using model averaging.

### Statistical analysis

We associated between social parameters and age of death, using linear regression. Because the age of death variable was not normally distributed, we calculated the P values for the regressions using randomization test [45]. We used regression residuals to control for the effects of group size and observation period. All least squares regression models were calculated using Permute! 3.4 (www.bio.umontreal.ca/Casgrain/en/labo/permute) and JMP (version 9, SAS Inc.).

## Results

Our community detection analysis identified two social groups for each year at each site (Fig. S1), except for the Arugot site during the years 2000 and 2009, in which additional groups comprising 3–4 animals each were detected. We excluded those four small groups from our analysis since their survival data were incomplete. The mean group size was 13.9±5.1 and the mean sequential change of group size to the following year was 27%±25. Group average non-zero association index values ranged between 0.174 and 0.369.

**Figure 2:**
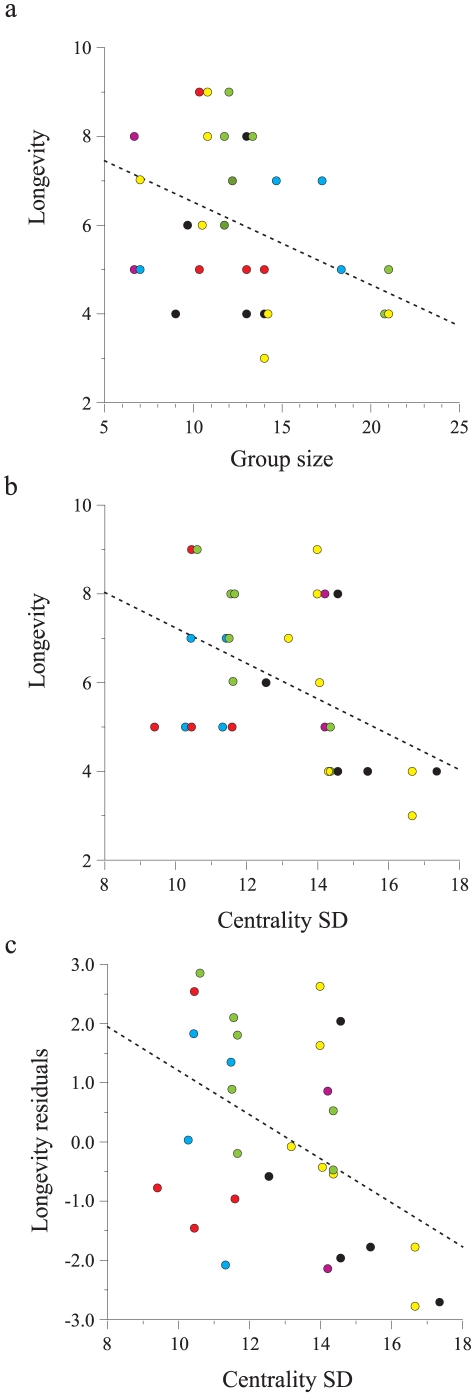
Longevity (age at death) of rock hyraxes as a function of group size (r^2^_34_ = 0.179, slope = −0.186, P = 0.014; a), centrality SD (r^2^_34_ = 0.201, slope = −0.399, P = 0.007; b), and centrality SD controlled for group size (r^2^_34_ = 0.212, slope = −0.371, P = 0.006; c). Colors denote social groups: green = group I 2000–2004, blue = group I 2007–2009, yellow = group S 2000–2004, black = group S 2007–2009, red = group C, purple = group W.

Centrality and its standard deviation (SD) varied considerably among social groups. In some groups members had similar level of centrality, whereas in other groups the differences in centrality among group members was substantial (e.g. Fig. 1). Utilizing a conservative approach, we examined social network data for 34 group members of known age and who died as adults. Thus, animals that were either solitary, died before reaching adulthood, or that were still alive when this analysis took place, were excluded. The mean change of group centrality SD during an adult's lifetime was 19.3%±9.9. We found that adult longevity (i.e. age at death) was negatively associated with group size; defined as the mean group size in the years the specific individual belonged to the group (regression by randomization test: r^2^_34_ = 0.179, slope = −0.186, P = 0.014, Fig. 2a). Longevity did not correlate with average group centrality (r^2^_34_ = 0.048, P = 0.220). However, longevity was significantly predicted by group centrality SD (regression by randomization test: r^2^_34_ = 0.201, slope = −0.399, P = 0.007, Fig. 2b), which was retained also after controlling for the effect of group size (r^2^_34_ = 0.212, slope = −0.371, P = 0.006, Fig. 2c). The contribution of sex to the model was insignificant (age at death: F_1,32_ = 0.03, P = 0.852; age at death controlled for group size: F_1,32_ = 0.64, P = 0.428). Centrality SD was independent of group size (r^2^_34_ = 0.006, P = 0.674).

To accommodate for possible dependency between group members, we added a group random effect to the regression model. This group variable assigned the known dead individuals to their social groups. In Arugot creek, we assigned the animals to four groups, two that were monitored during 2000–2004 and two during 2007–2009. This approach was justified because nearly all animals present during the earlier period were already gone in the later period (Group I: 5% overlap between periods, n = 58; Group S: 3% overlap between periods, n = 36), thus social groups were composed of different individuals during 2000–2004 and 2007–2009 (see example in Fig. 1). The regression model with the group effect accounted for 42.7% of the variance in longevity (F_6,27_ = 3.4, P = 0.0134). However, only centrality SD showed a significant effect on longevity controlled for group size (F_1,27_ = 14.6, P = 0.0007). The group random effect was insignificant (F_5,27_ = 2.0, P = 0.107). Furthermore, longevity, controlled for group size, did not correlate with intra group aggression (r^2^ = 0.02, F_1,27_ = 2.7, P = 0.110) or with individual aggression rate (r^2^ = 0.053, F_1,27_ = 1.5, P = 0.232).

Longevity did not correlate significantly with individual power or information centrality (r^2^_34_ = 0.03, P = 0.316 and r^2^_34_ = 0.03, P = 0.375, respectively). However, an inverse association was apparent, although not significant, between longevity and group power SD (regression by randomization test: r^2^_34_ = 0.091, slope = −1.00, P = 0.078) or group information centrality SD (regression by randomization test: r^2^_34_ = 0.098, slope = −4.95, P = 0.074). Distance based cohesion and network centralization did not correlate with longevity (r^2^_34_ = 0.11, P = 0.058 and r^2^_34_ = 0.06, P = 0.157, respectively), and distance based cohesion was independent of longevity even after controlling for group size and dependency (F_1,26_ = 0.3, P = 0.564).

**Figure 3:**
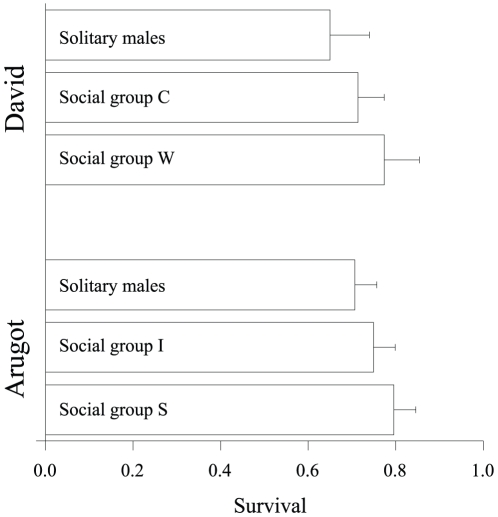
Yearly average mark-recapture survival estimates for social groups and solitary male rock hyraxes at Arugot and David sites, calculated by model averaging and the Barker model [**34**]. Error bars denote model SE.

Solitary male survival was constant over time, and significantly lower compared to group member's survival for both populations (randomization test: P = 0.054, Fig. 3, Table S2, Table S3), even though the difference in survival is small. This result was confirmed by the likelihood ratio test for Arugot (χ^2^_16_ = 67.568, P<0.0001) and David (χ^2^_7_ = 15.455, P = 0.03) sites. All groups showed age structure in survival, with a difference between first-year juveniles and adults (Table S2, Table S3). Recapture rate was high and did not vary with time in both populations (Table 1). Further details on the mark-recapture results are given in Text S1.

**Table 1:** Summary of group size, recapture probability (*P_p_*) and survival probability (*S_p_*) estimates for the Arugot and David sites.

	David			Arugot		
Group name	Group C	Group W	Male	Group I	Group S	Male
Number of years	8	8	8	10	10	10
Group size	5–18	6–10		7–21	7–15	
*P_p_*	0.828	0.828	0.828	0.891	0.891	0.891
*P_p_* SE	0.038	0.038	0.038	0.025	0.025	0.025
*S_p_*	0.774	0.714	0.650	0.796	0.749	0.707
*S_p_* SE	0.050	0.080	0.090	0.050	0.050	0.060

Recapture and survival estimates, averaged over years, were obtained using model averaging.

## Discussion

In this study we found that non-aggressive social interactions among hyraxes, expressed by social networks parameters, as well as group size, predict longevity. Members of smaller groups and members of groups that were more egalitarian lived longer. Specifically, we found that the variance in centrality within the group network (i.e. centrality SD) accounted for the differences found in hyrax longevity, even after the effect of group size was removed. Social groups differed greatly in their centrality SD (Fig. 1): some groups showed higher skew in centrality, with a few highly connected individuals, while other groups showed homogeneity in the way centrality is distributed across the network. A low variance in centrality may reflect a more socially stable and less stressful environment, thus enhancing the recognized benefits of group living [12]. Since controlling for group size did not change the association between longevity and centrality SD, the skew in network centrality is probably not an artifact of larger groups [46]. In this respect, our findings are novel by showing the potential role of centrality SD as a key predictor for longevity within social groups. It is interesting to note that although non significant, the group SD of the other two individual centrality measures we used (i.e. power and information centrality) also showed an inverse relationship with longevity. Conversely, individual measures of centrality could not predict longevity. These results show that it is not necessarily the most central individuals in the group that survive longer. Alternatively, variance in social associations within the group may negatively affect all group members. Our results add a new twist to the view presented by recent studies showing correlation between number of individual social associations and survival [11]–[14].

Why is it advantageous for a group to have an equally distributed centrality among its members? Perhaps the existence of a few dominant and highly connected individuals, which monopolize power in the network, leads to isolation of group members and leaves them vulnerable to predation. In a fragmented habitat such as the rock outcrops where hyrax live, and where the constant flow of information to all parts of a network is important, the social configuration of a dominance hierarchy may not be beneficial for the group members. Additionally, differences in rank and hierarchy steepness are known to affect the stress levels of animals in numerous societies [47]. Thus, one possible explanation to the lower longevity in hyrax groups where social inequality was observed is greater physiological stress, although this cause was not directly assessed. A stress related theory may also explain why individual centrality parameters could not predict longevity since in a more stressful group all members may be affected, thus a stressful group may impair the health of socially central individuals as well as that of less central ones. An alternative explanation is a possible association between inequality and aggression. Numerous wildlife studies have considered individual social rank, a measure based on agonistic interaction, as one having leading role on individual survival and fitness (e.g. [48], [49]). Furthermore, recent studies showed that agonistic relationships are a major factor determining fitness [50]. In our system, aggressive interactions did not affect longevity. One possible explanation to the lack of association between level of aggression and survival within hyrax society may be related to the rarity of this type of interaction. Similar patterns are observed in other species where hierarchy is highly transitive and the risk of injury possibly deters physical aggression [51]. Thus, how inequality within a social group leads to decrease in longevity is still an open question, which requires further research.

Beyond the importance of equality in social ties, our results provide an additional line of evidence for the adaptive value of group living: we show that solitary male hyraxes that were not socially attached to groups or had few connections (Fig. S1) also showed lower survival (Fig. 3). These results are in line with findings from mammal studies demonstrating the advantages of sociality (e.g. [1], [12]). The costs of solitary life among wildlife may include greater vulnerability to predators and competition for home ranges and resources. Juvenile male rock hyraxes are forced to disperse from their natal groups [15], [23]. To maximize their chances for survival, they are predicted to delay dispersal for as long as possible [52]. This may lead to conflicts with other group members, which our results predict would prefer to live in smaller groups, where survivorship is higher. The benefits that individuals obtain from living in larger groups have been widely demonstrated in the literature. Larger groups provide more opportunities for fusion-fission [53], are able to displace smaller groups from their territories, and may even kill some of the latter's members [54]. However, individuals in larger groups may be forced to cover longer distances while foraging, due to stronger competition for food [55], and communication with other group members may consequently become constrained. Furthermore, large number of members may reduce the quality of food resources and increase competition for sleeping places. Another disadvantage of large groups is the reduced intra-group relatedness, which could result in more conflicts within the group [56]. The disadvantage of larger groups in our study system may be manifested by our observations of females dispersing away from larger groups to join smaller ones (seven dispersal events from two social groups), while no females were observed dispersing from smaller to larger groups. Altogether, the negative correlation we found between group size and survival in rock hyrax contradicts group augmentation theories [54], which have been demonstrated in breeding systems with high reproductive skew [57].

Longevity is considered a key component in lifetime reproductive success [14], [58]. While reproductive fitness is a more commonly used measure [12], focusing on offspring survival, adult longevity may reflect on the animal's health and physiological condition. The notion that the structure of animal social networks might affect the survival probability of their adult members is novel. Interestingly, numerous studies on humans found an increased likelihood of survival for individuals with stronger social ties [59]. However, only a few animal studies have used the group level approach, as most similar work has focused on individual network variables. Our results demonstrate the significance of the social environment surrounding an animal to its well-being and survival. Social networks provide an excellent framework for hypothesis testing in this context. While ecological factors seem to be important in determining the survival of animals in a group [2], novel properties like the variation in centrality appears to play a larger role than thought before, providing exciting prospects for further research.

## Supporting Information

Figure S1 **Examples of two social networks for David (a) 2008 and Arugot 2009 (b) populations.** Social groups, following community detection results (see methods section), are delimited by dotted lines. Males and females are represented by empty and full circles, respectively. Line width between nodes is proportional to the association index values, representing strength of ties.
(EPS)

Table S1 **parameter combinations modeled with Barker models. Notation: t = time dependent, . = constant, 2a = 2 age classes, d_i_, d_j_ = group names, g = group, m = males.**
(DOC)

Table S2 **Summary results of the Barker model analysis of survival and recapture rates for the Arugot population between 2000 and 2009 in Ein Gedi, Israel.** Models highlighted in bold are the best-supported models in the candidate set. Additional parameters were previously modeled and kept constant: r(.)R(06- ./.)R′(06- ./.)F(g = 1, m-.)F′(g-., m = 0). See Table S1 for notation. Group names: d_i_ = I, d_j_ = S. Weight presented was calculated relative to the models tested in the table.
(DOC)

Table S3 **Summary results of the Barker model analysis of survival and recapture rates for the David population between 2002 and 2009 in Ein Gedi, Israel.** Models highlighted in bold are the best-supported models in the candidate set. Additional parameters were previously modeled and kept constant: r(t) R(06- ./.)R′(06- ./.)F(g-., m-.)F′(.). Group names: d_i_ = C, d_j_ = W. See Table S1 for notation. Weight presented was calculated relative to the models tested in the table.
(DOC)

Text S1 (DOC)

## References

[1] Krause J, Ruxton GD (2002). Living in groups.

[2] Alexander RD (1974). The evolution of social behavior.. Ann Rev Ecol Syst.

[3] Packer C, Scheel D, Pusey AE (1990). Why lions form groups – food is not enough.. Am Nat.

[4] Creel S, Creel NM (1998). Six ecological factors that may limit African wild dogs, *Lycaon pictus*.. Anim Conserv.

[5] Hodge SJ (2005). Helpers benefit offspring in both the short and long-term in the cooperatively breeding banded mongoose.. Proc R Soc B-Biol Sci.

[6] Mosser A, Packer C (2009). Group territoriality and the benefits of sociality in the African lion, *Panthera leo*.. Anim Behav.

[7] Clutton-Brock TH, Brotherton PNM, Russell AF, O'Riain MJ, Gaynor D (2001). Cooperation, control, and concession in meerkat groups.. Science.

[8] Clutton-Brock TH (2009). Structure and function in mammalian societies.. Philos T Roy Soc B.

[9] Hoogland JL (1983). Black-Tailed prairie dog coteries are cooperatively breeding units.. Am Nat.

[10] Randall JA, Rogovin K, Parker PG, Eimes JA (2005). Flexible social structure of a desert rodent, *Rhombomys opimus*: philopatry, kinship, and ecological constraints.. Behav Ecol.

[11] Silk JB, Alberts SC, Altmann J (2003). Social bonds of female baboons enhance infant survival.. Science.

[12] Silk JB (2007). The adaptive value of sociality in mammalian groups.. Philos Trans R Soc B-Biol Sci.

[13] Cameron EZ, Setsaas TH, Linklater WL (2009). Social bonds between unrelated females increase reproductive success in feral horses.. Proc Natl Acad Sci USA.

[14] Silk JB, Behhner JC, Bergman TJ, Crockford C, Engh AL (2010). Strong and consistent social bonds enhance the longevity of female baboons.. Curr Biol.

[15] Koren L (2000). Hyrax socialization: First evidence for a matriarchal society. M Sc Thesis.

[16] Koren L, Geffen E (2009). Androgens and social status in female rock hyraxes.. Anim Behav.

[17] Hoeck HN, Klein H, Hoeck P (1982). Flexible social organization in hyrax.. Z Tierpsychol.

[18] Koren L, Mokady O, Geffen E (2006). Elevated testosterone levels and social ranks in female rock hyrax.. Horm Behav.

[19] Hoeck HN (1982). Population dynamics, dispersal and genetic isolation in 2 species of hyrax (*Heteroyrax brucei* and *Procavia johnstoni*) on habitat islands in the Serengeti.. Z Tierpsychol.

[20] Krause J, Lusseau D, James R (2009). Animal social networks: An introduction.. Behav Ecol Sociobiol.

[21] Burnham KP, Anderson AE (2002). Model Selection and Multimodel Inference: a Practical Information-Theoretic Approach.

[22] Hoeck HN (1989). Demography and competition in hyrax – a 17 years study.. Oecologia.

[23] Altmann J (1974). Observational study of behavior - sampling methods.. Behaviour.

[24] Sale JB (1970). The behaviour of the resting rock hyrax in relation to its environment.. Zool Africana.

[25] Koren L, Mokady O, Geffen E (2008). Social status and cortisol levels in singing rock hyraxes.. Horm Behav.

[26] Barocas A (2010). Aspects of sociality in the rock hyrax *Procavia capensis*: social structure and factors predicting longevity. M Sc Thesis.

[27] Croft DP, James R, Krause J (2008). Exploring animal social networks.

[28] Estes RP (1991). The behavior guide to African mammals, including hoofed mammals, carnivores, primates.

[29] Whitehead H (2009). SOCPROG programs: Analysing animal social structures.. Behav Ecol Sociobiol.

[30] Bejder L, Fletcher D, Brager S (1998). A method for testing association patterns of social animals.. Anim Behav.

[31] Palla G, Derenyi I, Farkas I, Vicsek T (2005). Uncovering the overlapping community structure of complex networks in nature and society.. Nature.

[32] Adamcsek B, Palla G, Farkas IJ, Derenyi I, Vicsek T (2006). CFinder: Locating cliques and overlapping modules in biological networks.. Bioinformatics.

[33] Borgatti SP, Everett LG, Freeman LC (2002). Ucinet for Windows: Software for social network analysis.

[34] Barrat A, Barthelemy M, Pastor-Satorras R, Vespignani A (2004). The architecture of complex weighted networks.. Proc Natl Acad Sci USA.

[35] Bonacich P (1987). Power and centrality: A family of measures.. Am J Sociol.

[36] Stephenson KA, Zelen M (1989). Rethinking centrality: Methods and examples.. Soc Networks.

[37] Doreian P (1974). On the connectivity of social networks.. J Math Sociol.

[38] Scott J (1991). Social network analysis: A handbook.

[39] White GC, Burnham KP (1999). Program MARK: Survival estimation from populations of marked animals.. Bird Study.

[40] Barker RJ (1997). Joint modeling of live-recapture, tag-resight, and tag-recovery data.. Biometrics.

[41] Hall AJ, McConnell BJ, Barker RJ (2001). Factors affecting first-year survival in grey seals and their implications for life history strategy.. J Anim Ecol.

[42] Anderson DR, Burnham KP, White GC (1994). AIC model selection in overdispersed capture-recapture data.. Ecology.

[43] Lebreton JD, Burnham KP, Clobert J, Anderson DR (1992). Modeling survival and testing biological hypotheses using marked animals – a unified approach with case studies.. Ecol Monogr.

[44] Cooch EG, White GC (2001). A Gentle Introduction.. http://www.phidot.org/software/mark/docs/book.

[45] Manly BFJ (1994). Randomization and Monte Carlo methods in biology.

[46] James R, Croft DP, Krause J (2009). Potential banana skins in animal social network analysis.. Behav Ecol Sociobiol.

[47] Sapolsky RM (2005). The influence of social hierarchy on primate health.. Science.

[48] Schubert KA, Mennill DJ, Ramsay SM, Otter KA, Boag PT (2007). Variation in social rank acquisition influences lifetime reproductive success in black-capped chickadees.. Biol J Linn Soc.

[49] Hollister-Smith JA, Poole JH, Archie EA, Vance EA, Georgiadis NJ (2007). Age, musth and paternity success in wild male African elephants, *Loxodonta africana*.. Anim Behav.

[50] Lea AJ, Blumstein DT, Wey TW, Martin JGA (2011). Heritable victimization and the benefits of agonistic relationships.. Proc Natl Acad Sci USA.

[51] Wittemyer G, Getz MW (2007). Hierarchical dominance structure and social organization in African elephants, *Loxodonta Africana*.. Anim Behav.

[52] Kokko H, Johnstone RA (1999). Social queuing in animal societies: a dynamic model of reproductive skew.. Proc R Soc B.

[53] Ramos-Fernandez G, Boyer D, Aureli F, Vick LG (2009). Association networks in spider monkeys (*Ateles geoffroyi*).. Behav Ecol Sociobiol.

[54] Clutton-Brock T (2002). Breeding together: Kin selection and mutualism in cooperative vertebrates.. Science.

[55] Wrangham RW, Gittleman JL, Chapman CA (1993). Constraints on group size in primates and carnivores – population density and day range as assays of exploitation.. Behav Ecol Sociobiol.

[56] Aviles J, Fletcher JA, Cutter AD (2004). The kin composition of social groups: Trading group size for degree of altruism.. Am Nat.

[57] Clutton-Brock TH, Gaynor D, McIlrath GM, Maccoll ADC, Kansky R (1999). Predation, group size and mortality in a cooperative mongoose, *Suricata suricatta*.. J Anim Ecol.

[58] Clutton-Brock TH (1988). Reproductive Success: studies of individual variation in contrasting breeding systems.

[59] Holt-Lunstad J, Smith TB, Layton JB (2010). Social Relationships and Mortality Risk: A Meta-analytic Review.. PLoS Med.

