# Supplementary material for: Variance in Centrality within Rock Hyrax Social Networks Predicts Adult Longevity
Source: PLoS One. 2011 Jul 27;6(7):e22375. doi: 10.1371/journal.pone.0022375 (PMC3144894; doi:10.1371/journal.pone.0022375)
Supplement: Table S1 — parameter combinations modeled with Barker models. Notation: t = time dependent, . = constant, 2a = 2 age classes, di, dj = group names, g = group, m = males. (DOC) [file pone.0022375.s002.doc]

**Table S1**: parameter combinations modeled with Barker models. Notation: t=time dependent, .=constant, 2a= 2 age classes, d_i_, d_j_ =group names, g=group, m=males.

| Model description | Model Name |
| --- | --- |
| Basic model | S(g*t)p(t)r(t)R(t)R’(t)F(t)f’(t) |
| Survival  Age structure for each group, with time dependence for pups, constant for males.  Age structure for each group, no time dependence, constant for males.  Constant | S(d_i_ - 2a- t/. d_j_ - 2a-t/., m-.)  S(d_i_ -2a- ./. d_j_- 2a-./., m-.)  S(.) |
| Recapture  Time dependent  Constant | p(t)  p(.) |
| Dead Recovery  Time dependent  Constant | r(t)  r(.) |
| Resighting  Time dependent  Constant among years except 2006 (no observations)  Constant | R(t)  R(all, 06’ - ./0)  R(.) |
| Resighting before dead recovery  Time dependent  Constant among years, except 2006 (no observations)  Constant | R’(t)  R’(all, 06’ - ./0)  R’(.) |
| Fidelity  Time dependent  Group type effect  Constant | F(t)  F(g-., m-.)  R(.) |
| Return parameter  Time dependent  Group type effect  Constant | F’(t)  F’(g-., m-.)  F’(.) |
