# Supplementary material for: Variance in Centrality within Rock Hyrax Social Networks Predicts Adult Longevity
Source: PLoS One. 2011 Jul 27;6(7):e22375. doi: 10.1371/journal.pone.0022375 (PMC3144894; doi:10.1371/journal.pone.0022375)
Supplement: Table S2 — Summary results of the Barker model analysis of survival and recapture rates for the Arugot population between 2000 and 2009 in Ein Gedi, Israel. Models highlighted in bold are the best-supported models in the candidate set. Additional parameters were previously modeled and kept constant: r(.)R(06- ./.)R′(06- ./.)F(g = 1, m-.)F′(g-., m = 0). See Table S1 for notation. Group names: di = I, dj = S. Weight presented was calculated relative to the models tested in the table. (DOC) [file pone.0022375.s003.doc]

**Table S2**: Summary results of the Barker model analysis of survival and recapture rates for the Arugot population between 2000 and 2009 in Ein Gedi, Israel. Models highlighted in bold are the best supported models in the candidate set. Additional parameters were previously modeled and kept constant: r(.)R(06- ./.)R’(06- ./.)F(g=1, m-.)F’(g-., m=0). See table S1 for notation. Group names: d_i_=Isiim, d_j_=Sukot. Weight presented was calculated relative to the models tested in the table.

|  | S | p | ∆QAICc | QAICc weight | No. Parameters | Deviance |
| --- | --- | --- | --- | --- | --- | --- |
| Modeling of recapture rates | S(t) | p(t) | 13.218 | 0 | 25 | 324.299 |
|  |  | p(d_i_-2a d_j_ -2a m-.) | 2.149 | 0.16 | 21 | 322.560 |
|  |  | p(2a) | 0.374 | 0.38 | 18 | 317.960 |
|  |  | **p(.)** | 0 | **0.46** | **17** | **320.435** |
| Modeling of survival in groups | **S(**d_i_ **-2a t/.** d_j_ **-2a ./. m-.)** | **p(.)** | **0** | **0.49** | **21** | **302.936** |
|  | S(g-2a., m-.) |  | 1.337 | 0.25 | 10 | 327.666 |
|  | S(d_i_ -2a ./. d_j_ -2a ./. m-.) |  | 1.580 | 0.22 | 12 | 323.468 |
|  | S(d_i_ -2a t/. d_j_ -2a t/. m-.) |  | 5.610 | 0.02 | 26 | 297.787 |
|  | S(d_i_ -2a ./. d_j_ -2a t/. m-.) |  | 6.895 | 0.02 | 17 | 318.319 |
| Modeling of male survival | **S(**d_i_ **-2a t/.** d_j_ **-2a ./. m-.)** | **p(.)** | **0** | **0.99** | **21** | **302.936** |
|  | S(d_i_ -2a t/. d_j_ -2a ./. m-t) |  | 14.479 | 0 | 30 | 297.298 |
