# Supplementary material for: Variance in Centrality within Rock Hyrax Social Networks Predicts Adult Longevity
Source: PLoS One. 2011 Jul 27;6(7):e22375. doi: 10.1371/journal.pone.0022375 (PMC3144894; doi:10.1371/journal.pone.0022375)
Supplement: Text S1 — (DOC) [file pone.0022375.s005.doc]

**Text S1**

A total of 285 animals, captured over 10 years, were included in the analysis. 168 of them were captured in the Arugot research area, and 117 in the David gorge. A total of 119 animals were captured as pups from four social groups, and 110 were aged as juveniles or adults at capture time. In addition, 56 solitary males were captured and not assigned to any group. Since animals were doubly marked, with either earrings or collars and microchips, mark loss was not significant (n=5). Group size varied over the research years between 5 and 21(Table 2). Male proportion was usually low, with values ranging between 0.07 and 0.33, with an unusual number of males in both the Arugot groups in the 2000 season (Isiim=0.476, Sukot=0.429).

**Mark-recapture Results.** The best support in the Arugot population was for constant area fidelity (F) over time. F differed between groups and solitary males (for groups F=1, for males F=0.93). Return probability (F’) was constant over time in groups (F’=0.45), and males (F’=0). In the David area, results were similar for the fidelity parameter, which differed between groups and solitary males (for groups F=0.966, for males F=0.747). Return parameter in David was constant, F’=0 Table S4, S5 )

Similar results were obtained for probability of resighting (R) and probability of resighting before dead recovery (R’). Since no observations were available for 2006, we built models with constant resighting and resighting before dead recovery probabilities for all occasions and different probabilities for 2006. According to the best supported models, R and R’ were constant in Arugot (R=0.979, R’=1) and David (R=0.975, R’=1). In both populations, R and R’ for the 2006 season were fixed at 0.

Probability of dead recovery (r) was constant in Arugot (r=0.019), and showed time variation in David (range between 0 and 0.57). In David recapture probability was constant in time and there was no difference between age classes (∆QAICc=1.431, p=0.828, Table 3). Recapture probability in Arugot was found to be high and constant (p=0.891), with slightly better support than an age dependent model (∆QAICc=0.374). No significant difference in recapture between groups was found (Table S2, S3).

The best supported model for adult survival in groups in Arugot showed age structure for both groups, with time variation for pup survival in the Isiim group, constant pup and adult survival in the Sukot group, and constant male survival. It was better supported (∆QAICc=1.337) than the next best model, which showed no difference between groups(Table S3). In the David population, the best supported model showed constant survival over time for both pups and adults in groups, without difference between groups (Table S4). The next best supported model showed time variation for pups in the Window group, and constant survival in other age classes (∆QAICc=2.817). Models with time dependent male survival, when compared with constant male survival, showed significantly lower support (David: ∆QAICc=14.479, Arugot: ∆QAICc=2.14).
